# The INDEPTH standard population for low- and middle-income countries, 2013

**DOI:** 10.3402/gha.v7.23286

**Published:** 2014-03-27

**Authors:** Osman Sankoh, David Sharrow, Kobus Herbst, Chodziwadziwa Whiteson Kabudula, Nurul Alam, Shashi Kant, Henrik Ravn, Abbas Bhuiya, Le Thi Vui, Timotheus Darikwa, Margaret Gyapong, Momodou Jasseh, Nguyen Chuc Thi Kim, Salim Abdullah, Amelia Crampin, John Ojal, Seth Owusu-Agyei, Frank Odhiambo, Mark Urassa, Kim Streatfield, Masaaki Shimada, Charfudin Sacoor, Donatien Beguy, Karim Derra, George Wak, Valerie Delaunay, Ali Sie, Abdramane Soura, Diadier Diallo, Siswanto Wilopo, Honorati Masanja, Bassirou Bonfoh, Suparat Phuanukoonnon, Samuel J. Clark

**Affiliations:** 1 INDEPTH Network, Accra, Ghana; 2 School of Public Health, University of the Witwatersrand, Johannesburg, South Africa; 3 Faculty of Public Health, Hanoi Medical University, Hanoi, Vietnam; 4 University of Washington, Seattle, USA; 5 Africa Centre, Kwa-Zulu Natal, South Africa; 6 MRC/Wits Rural Public Health and Health Transitions Research Unit (Agincourt), School of Public Health, Faculty of Health Sciences, University of the Witwatersrand, Johannesburg, South Africa; 7 AMK HDSS, Dhaka, Bangladesh; 8 Ballabgarh HDSS, Ballabgarh, India; 9 Bandim HDSS, Bissau, Guinea Bissau; 10 Chakaria HDSS, Dhaka, Bangladesh; 11 Chililab HDSS, Chililab, Vietnam; 12 Dikgale HDSS, Polokwane, South Africa; 13 Dodowa HDSS, Accra, Ghana; 14 Farafanni HDSS, Farafanni, The Gambia; 15 Filabavi HDS, Filabavi, Vietnam; 16 Ifakara HDSS, Dar es Salaam, Tanzania; 17 Karonga HDSS, Karonga, Malawi; 18 Kilifi HDSS, Kilifi, Kenya; 19 Kintampo HDSS, Kintampo, Ghana; 20 Kisumu HDSS, Kisumu, Kenya; 21 Magu HDSS, Magu, Tanzania; 22 Matlab HDSS, Dhaka, Bangladesh; 23 Mbita HDSS, Mbita, Kenya; 24 Manhica HDSS, Manhica, Mozambique; 25 Nairobi HDSS, Nairobi, Kenya; 26 Nanoro HDSS, Nanoro, Burkina Faso; 27 Navrongo HDSS, Navrongo, Ghana; 28 Niakhar HDSS, Niakhar, Senegal; 29 LPED, IRD/Aix-Marseille Université, Aix-Marseille, France; 30 Nouna HDSS, Nouna, Burkina Faso; 31 Ouagadougou HDSS, Ouagadougou, Burkina Faso; 32 Oubritenga HDSS, Oubritenga, Burkina Faso, PATH, Dakar, Senegal; 33 Purworejo HDSS, Purworejo, Indonesia; 34 Rufiji HDSS, Rufiji, Tanzania; 35 Taabo HDSS, Taabo, Cote d'Ivoire; 36 Wosera HDSS, Wosera, Papua New Guinea; 37 ALPHA Network, London, UK

**Keywords:** crude death rate, age-specific mortality rate, age-standardized crude death rate, demography, standardized age structure, low- and middle-income countries

## Abstract

Crude rates such as the crude death rate are functions of both the age-specific rates and the age composition of a population. However, differences in the age structure between two populations or two time periods can result in specious differences in the corresponding crude rates making direct comparisons between populations or across time inappropriate. Therefore, when comparing crude rates between populations, it is desirable to eliminate or minimize the influence of age composition. This task is accomplished by using a standard age structure yielding an age-standardized rate. This paper proposes an updated International Network for the Demographic Evaluation of Populations and Their Health (INDEPTH) standard for use in low- and middle-income countries (LMICs) based on newly available data from the health and demographic surveillance system site members of the INDEPTH network located throughout Africa and southern Asia. The updated INDEPTH standard should better reflect the age structure of LMICs and result in more accurate health indicators and demographic rates. We demonstrate use of the new INDEPTH standard along with several existing ‘world’ standards and show how resulting age-standardized crude deaths rates differ when using the various standard age compositions.

In demography and related fields, crude rates such as the crude death rate (CDR) are functions of both the age-specific rates and the age composition of a population, that is, the proportional distribution of people across age. However, as usually computed, these crude rates fail to account for age variation. Consequently, differences in the age structure between two populations or two time periods can result in specious differences in the corresponding crude rates making direct comparisons between populations or across time inappropriate. Therefore, when comparing crude rates between populations, it is desirable to eliminate or minimize the influence of age composition (1, pp. 24–28). This task is accomplished by using a standard age structure yielding an age-standardized rate.

In the case of death rates, the age-standardized crude death rate (ASCDR) is an age-weighted average of the age-specific mortality rates for the populations to be compared. The ASCDR represents a summary measure that reflects the rate of death assuming the populations for comparison have identical age structures.

Numerous international standards have been proposed in the search for a ‘world’ standard (see (2) for a brief review of the history of these standards), including two widely adopted standards. Epidemiologist Mitsuo Segi proposed a ‘world’ standard based on data representing 46 countries (3), while the World Health Organization (WHO) constructed a new standard in 2001 based on the average age structure of the world during the period 2000–2025 as estimated in the World Population Prospects 1998 (2). Compared to the Segi standard, the WHO standard has fewer children proportionally and a greater proportion of adults aged 70+. The International Network for the Demographic Evaluation of Populations and Their Health (INDEPTH) also proposed a standard in 2002 specifically for low- and middle-income countries (LMICs) based on data from 17 health and demographic surveillance system (HDSS) sites located throughout Africa and southeast Asia (4, 5). Since that time, the availability of high-quality data from INDEPTH member sites has increased to 32 HDSSs. The greater amount of available data, representing a wider range of contexts in LMICs, calls for an updated INDEPTH standard.

Although the choice of a standard is ultimately arbitrary, use of varying standards can change comparisons between populations considerably (1, 2, 5). For instance, using an ‘older’ structure like the 2001 WHO standard will weigh more heavily the age-specific rates at more advanced ages. Furthermore, both the WHO and Segi standards reflect to a greater extent populations with relatively low fertility and mortality giving more weight to the middle years of life. Applying these standards to ‘younger’ populations (where a greater proportion of their total population is concentrated at the youngest ages) can misstate the true level of mortality. To produce reliable indicators and better understand the age structure of some LMICs, a standard for these settings is necessary.

In this paper, we propose an updated INDEPTH standard for use in LMICs based on newly available data from the HDSS site members of the INDEPTH network located throughout Africa (eastern, western, southern) and southern Asia. The updated INDEPTH standard should better reflect the age structure of LMICs.^1^

## The INDEPTH standard for LMICs

The INDEPTH standard is constructed from a database containing 332 single year age distributions of observed person-years. These data represent 32 HDSSs with varying years of observation from 1983 to 2011. The HDSS data availability along with the total person-years contributed from each HDSS is summarized in Table 1.

**Table 1:** Summary of life tables used to calculate 2013 INDEPTH age standard showing HDSS site name, country, years of available data and total person-years contributed to the 2013 INDEPTH standard

HDSS	Country	Years	Person-years
Africa Centre	South Africa	2000–2009	650106.2
Agincourt	South Africa	1994–2008	1021000.9
AMK-Abhoynagar	Bangladesh	1983–2005	561262.7
AMK-Mirsarai	Bangladesh	1995–2005	353099.1
Ballabgarh	India	1993–2009	1314106.6
Bandim	Guinea Bissau	2000–2004	196673.9
Chakaria	Bangladesh	2005–2009	234267.3
Chililab	Vietnam	2005–2011	372372.0
Dikgale	South Africa	1996–2003	63873.5
Dodowa	Ghana	2007–2009	323766.6
Farafenni	The Gambia	1993–2008	419199.7
Filabavi	Vietnam	1999–2009	509135.9
Ifakara	Tanzania	1997–2010	1042163.3
Karonga	Malawi	2003–2008	174361.3
Kilifi	Kenya	2001–2008	1586821.3
Kintampo	Ghana	2005–2008	491974.0
Kisumu	Kenya	2006–2008	536367.8
Magu	Tanzania	1995–2008	362446.2
Manhica	Mozambique	1997–2011	959583.6
Matlab	Bangladesh	1983–2008	5473341.7
Mbita	Kenya	2009–2011	196635.9
Nairobi	Kenya	2003–2009	398507.6
Nanoro	Burkina Faso	2009–2010	92398.6
Navrongo	Ghana	1996–2009	1966614.9
Niakhar	Senegal	1990–2011	700855.3
Nouna	Burkina Faso	1992–2008	860827.4
Ouagadougou	Burkina Faso	2009–2011	219085.2
Oubritenga	Burkina Faso	1993–2003	1049908.4
Purworejo	Indonesia	1995–2005	616580.1
Rufiji	Tanzania	1999–2010	942420.6
Taabo	Côte d'Ivoire	2009–2011	103949.1
Wosera	Papua New Guinea	1999–2004	72879.9
		Total	23866586.4

INDEPTH=International Network for the Demographic Evaluation of Populations and Their Health; HDSS=health and demographic surveillance system.

These data have been screened for consistency and plausibility before inclusion in the final data set. For each HDSS, we first plotted the age-specific deaths and person-years by year and removed HDSS-years that were out of trend given historical rates at that HDSS or an entire HDSS's data series if the data were demographically implausible. We next calculated a life table for each HDSS-year and plotted the annual age-specific mortality rates as well as the annual trend in child mortality (_5_q_0_ or the probability a newborn will die before his or her fifth birthday), adult mortality (_45_q_15_ or the probability that a 15-year-old will die before reaching his or her 60th birthday), and life expectancy at birth and removed any out-of-trend years. Data inclusion for each HDSS was also confirmed with an HDSS representative who could verify the trend in mortality data with information about conditions at the HDSS or insights into data collection issues or protocol at the HDSS (e.g. it may be known to a site representative that child deaths are systematically underreported). Of the 37 HDSSs that submitted mortality data, five were screened out entirely after an HDSS representative verified that data were incomplete or unreliable for all years in an HDSS time series. Of the remaining 32 HDSSs, 14 had a portion of their data series removed. Either the first or final year of data from these HDSSs time series was discarded if the person-years or deaths were lower than expected because the data were incomplete for recent years or suitable data collection protocols had not been solidified in early years. After screening inapplicable data, the INDEPTH collection contains approximately 23.9 million person-years of observation.

**Fig. 1:**
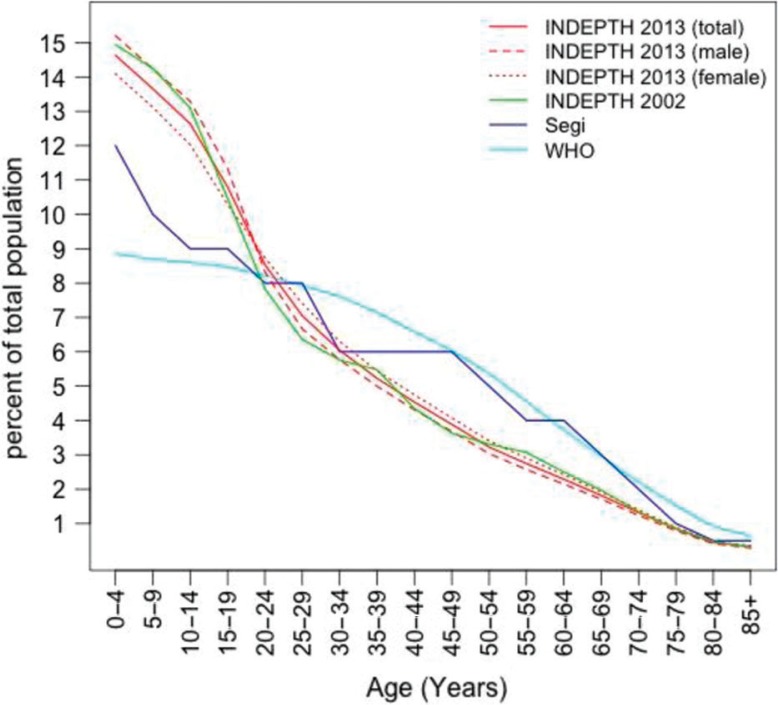
Standard population age structure for INDEPTH 2013, also showing INDEPTH 2002, Segi and WHO standards.

The INDEPTH standard is a weighted average of the proportion of person-years lived in each age interval (ages 0–4, 5–9, 10–14, …, 85+)^2^ for each of the 332 single year life tables. Since HDSSs vary greatly in size, each of the 332 sets of age-specific person-years is weighted by the proportion of total person-years contributed for that HDSS-year to the total number of person-years in the entire dataset. The 2013 INDEPTH standard and the 2002 INDEPTH standard are plotted in Fig. 1 along with the Segi and WHO world standards (numerical values can also be found in Table 2). Compared to the 2002 INDEPTH standard, the 2013 standard has a slightly greater proportion of the population between ages 15–35 and somewhat less in ages under 10. The 2013 INDEPTH standard also features larger proportions of the population in younger age intervals relative to the WHO and Segi standards reflecting the relatively higher fertility and mortality for the INDEPTH HDSS sites compared to the populations from which the Segi and WHO standards were derived. Finally, population age composition is largely the result of historical and prevailing fertility and mortality rates but migration patterns can also influence the age structure at HDSSs. Migration affects age structures in various ways, but the predominant effect is to remove working age men, and to a lesser extent women. Occasionally, women migrate with very young children, so a small effect for the youngest ages is sometimes present (5, 6). This new INDEPTH standard should better reflect the overall state of mortality in LMICs by weighting more heavily the age-specific mortality rates below age 25 and reducing the weight given to mortality rates above age 35.

**Table 2:** Standard population age structure (% of total population in each age interval) for INDEPTH 2013 (total, male, and female), INDEPTH 2002, Segi, and WHO

Age	INDEPTH (2013)	INDEPTH (2013, male)	INDEPTH (2013, female)	INDEPTH (2002)	Segi	WHO
*0*	*3.07*	*3.19*	*2.95*			
*1–4*	*11.56*	*12.01*	*11.14*			
0–4	14.63	15.20	14.09	14.94	12.00	8.86
5–9	13.66	14.23	13.12	14.25	10.00	8.69
10–14	12.64	13.29	12.03	13.10	9.00	8.60
15–19	10.80	11.34	10.29	10.46	9.00	8.47
20–24	8.54	8.32	8.74	7.83	8.00	8.22
25–29	7.05	6.66	7.40	6.36	8.00	7.93
30–34	6.04	5.77	6.30	5.76	6.00	7.61
35–39	5.23	5.00	5.44	5.48	6.00	7.15
40–44	4.54	4.31	4.75	4.35	6.00	6.59
45–49	3.89	3.68	4.08	3.63	6.00	6.04
50–54	3.23	3.03	3.41	3.31	5.00	5.37
55–59	2.74	2.57	2.90	3.07	4.00	4.55
60–64	2.29	2.14	2.42	2.50	4.00	3.72
65–69	1.82	1.71	1.92	1.97	3.00	2.96
70–74	1.32	1.24	1.40	1.34	2.00	2.21
75–79	0.84	0.79	0.89	0.85	1.00	1.52
80–84	0.44	0.41	0.47	0.47	0.50	0.91
85+	0.32	0.29	0.35	0.32	0.50	0.63

INDEPTH=International Network for the Demographic Evaluation of Populations and Their Health; WHO=World Health Organization.

Table 3 shows the CDR calculated as the total number of deaths divided by the total person-years for the most recent 5-year period of each HDSS^3^ along with the ASCDRs using the two INDEPTH, Segi, and WHO standards. Each of the four columns showing the ASCDR has a column to the right indicating the rank order for that ASCDR. Those ASCDR ranks that differ in order from the 2013 standard ranks are shown in red text. Compared to using the 2002 standard, the 2013 INDEPTH standard produces marginally smaller ASCDRs, an average difference of −0.168 across all HDSS, reflecting the slightly smaller proportion of individuals below age 15 and larger proportions in age groups 20–45. In the opposite direction, the Segi and WHO standards, which reflect generally ‘older’ populations, typically overestimate the level of mortality by giving too much weight to the relatively higher mortality rates in the middle ages and too little weight to the mortality rates below age 20.

**Table 3:** Crude death rate (CDR=total deaths/total PYs) per 1,000 and age-standardized death rates calculated with the INDEPTH 2013, 2002, Segi, and WHO standards

		ASCDR							
		
HDSS	CDR	INDEPTH 2013	Rank	INDEPTH 2002	Rank	Segi	Rank	WHO	Rank
Filabavi	5.9	3.5	1	3.6	1	4.5	1	5.2	1
Chililab	5.8	3.7	2	3.7	2	4.7	2	5.4	2
AMK-Abhoynagar	5.7	5.9	3	6.1	3	7.3	3	8.0	3
Matlab	6.8	6.3	4	6.4	4	7.9	6	8.9	11
Purworejo	11.4	6.3	5	6.5	5	8.2	4	9.4	8
Niakhar	6.6	6.5	6	6.6	6	7.8	8	8.7	6
Ouagadougou	4.7	6.6	7	6.7	7	8.2	11	9.0	4
Chakaria	6.1	6.7	8	6.9	8	8.1	7	8.7	7
Dodowa	7.1	6.8	9	6.9	9	8.6	5	9.4	5
Dikgale	7.9	6.8	10	6.9	10	8.9	9	10.2	9
Mbita	6.1	7.0	11	7.1	11	8.2	12	8.6	12
AMK-Mirsarai	7.0	7.0	12	7.2	12	8.7	10	9.6	15
Kilifi	6.6	7.5	13	7.7	13	9.3	13	10.1	13
Ballabgarh	6.9	7.6	14	7.8	14	9.3	14	10.2	10
Rufiji	9.7	8.1	15	8.3	15	9.4	15	9.8	14
Kintampo	7.8	8.1	16	8.3	16	9.7	16	10.3	16
Farafenni	7.8	8.6	17	8.8	17	10.7	18	11.6	18
Ifakara	8.9	9.0	18	9.1	18	10.3	17	10.8	21
Magu	8.3	9.3	19	9.4	19	11.0	21	11.7	17
Nairobi	7.6	9.6	20	9.7	20	11.2	19	11.7	19
Taabo	8.4	9.7	21	9.8	21	11.0	20	11.4	20
Karonga	9.4	9.9	22	10.1	22	11.9	22	12.9	26
Navrongo	10.9	10.1	23	10.4	23	12.3	23	13.1	22
Nouna	10.7	10.9	24	11.2	24	12.5	24	13.1	24
Agincourt	11.9	12.2	25	12.3	25	15.0	26	16.0	23
Oubritenga	13.5	12.2	26	12.5	26	12.8	25	12.7	25
Wosera	10.4	12.4	27	12.8	27	15.8	27	16.8	27
Nanoro	12.3	13.3	28	13.5	28	17.2	28	19.8	30
Africa Centre	13.7	14.6	29	14.7	29	18.2	29	19.7	29
Manhica	15.2	15.4	30	15.5	30	18.5	30	19.5	28
Kisumu	18.2	17.9	31	18.0	31	20.2	31	21.0	31
Bandim	17.0	20.8	32	21.3	32	25.0	32	26.0	32

The ‘rank’ column to the right of each ASCDR shows the relative rank when using a given age-standard with ranks differing from the 2013 standard shown in red font. HDSS are ordered according to the ASCDR using the 2013 INDEPTH standard. ASCDR=age-standardized crude death rate; HDSS=health and demographic surveillance system; CDR=crude death rate; INDEPTH=International Network for the Demographic Evaluation of Populations and Their Health; WHO=World Health Organization.

Of course, the primary purpose of the age standard is comparison of populations with different age structures. If we examine the differences resulting from the different standards across HDSS by comparing the ‘rank’ columns, substantial differences emerge in not only the amount of difference between standardized rates but also the direction of that difference (1). For example, the Matlab HDSS is ranked among the lowest mortality HDSS at number 4 using the INDEPTH standards, but occupies the 6th and 11th spots using the Segi and WHO standards respectively. The larger ASCDR for Matlab using the Segi and WHO standards results from the larger weights at older ages where mortality rates are highest. Matlab's age structure is younger than the Segi or WHO and thus requires greater weight to the relatively lower mortality rates at younger ages to more closely estimate the level of mortality. Even in places where adult mortality rates are relatively high due to HIV/AIDS-related deaths such as in Agincourt, the ASCDRs are larger when using the Segi or WHO standards, which heavily weight higher mortality rates at the oldest ages. For many of these high adult mortality HDSS, the ‘younger’ INDEPTH standard yields ASCDRs that more closely approximate the CDRs as more weight is given to low childhood mortality rates.

## INDEPTH standards for sub-Saharan Africa and Asia

For comparing LMIC populations across continents, a single standard for LMICs is useful but in the event of comparing just sub-Saharan African (SSA) populations in SSA, it may be more intuitive to use a standard simply for SSA. In addition, African and Asian HDSSs differ considerably in the proportion of their populations aged less than 15 years. To this end, we have calculated separate standard age structures using HDSSs from SSA and those from Asia. These additional standards can be found in Table 4, which disaggregates the 2013 INDEPTH standard for LMICs by region and recalculates age standards using HDSSs from SSA and Asia separately. Of the total 332 life tables used to calculate the INDEPTH 2013 standard, 217 (65%) are from HDSSs in SSA.

**Table 4:** INDEPTH age standards for SSA and Asia

	SSA			Asia		
		
Age	Total	Male	Female	Total	Male	Female
*0*	*3.46*	*3.63*	*3.31*	*2.47*	*2.54*	*2.41*
*1–4*	*12.79*	*13.37*	*12.24*	*9.72*	*10.00*	*9.45*
0–4	16.25	17.00	15.55	12.19	12.54	11.86
5–9	14.74	15.46	14.07	12.02	12.42	11.65
10–14	13.13	13.94	12.37	11.90	12.32	11.51
15–19	10.81	11.55	10.13	10.78	11.02	10.55
20–24	8.41	8.19	8.61	8.72	8.51	8.92
25–29	6.90	6.53	7.25	7.26	6.87	7.64
30–34	5.73	5.47	5.97	6.51	6.21	6.80
35–39	4.76	4.50	5.00	5.93	5.73	6.12
40–44	4.03	3.71	4.31	5.31	5.20	5.41
45–49	3.44	3.12	3.74	4.56	4.52	4.59
50–54	2.86	2.57	3.13	3.78	3.73	3.83
55–59	2.45	2.20	2.68	3.19	3.13	3.24
60–64	2.03	1.81	2.24	2.68	2.65	2.71
65–69	1.65	1.46	1.83	2.07	2.08	2.06
70–74	1.22	1.08	1.36	1.47	1.48	1.45
75–79	0.81	0.72	0.89	0.89	0.89	0.89
80–84	0.44	0.39	0.47	0.46	0.44	0.47
85+	0.35	0.30	0.39	0.28	0.26	0.29

INDEPTH=International Network for the Demographic Evaluation of Populations and Their Health; SSA=sub-Saharan Africa.

Compared to the overall 2013 INDEPTH standard, the region-specific standard for SSA reflects the relatively higher fertility and mortality of the HDSSs in this region. Roughly 44% of the total average person-years lived under age 15 for the SSA life tables in this collection compared to 41% for the entire dataset. The standard based solely on data from HDSSs in Asia contains approximately 36% of the total person-years lived in the under-15 age groups.

## Discussion

Removing or minimizing the effect of variation in age structure allows one to compare various summary demographic rates and health metrics between populations and across time. Choice of a standard is arbitrary, but choosing a standard with inappropriate age-weights can result in inaccurate estimates of the true level of mortality. We propose a new INDEPTH standard that mirrors the age structure of many LMICs and can produce more dependable mortality and health indicators.

Although the INDEPTH 2013 standard more closely reproduces the age composition of LMICs, a few cautionary notes are appropriate. HDSS data are overwhelmingly rural and to the extent that the age composition of urban populations in these countries differs from rural populations, the 2013 INDEPTH age standard will embody that difference. If urban populations are ‘older’, use of this standard may underestimate age-standardized mortality rates for example. Likewise, as is clear from Table 1, these data are largely contemporary. Just 28 of the 332 HDSS-years included in this standard are from prior to 1990 and the modal year of observation for this dataset is 2003 (n^2003^=46). Thus, the standard presented in this paper may not be appropriate for standardization of historical rates.

## Footnotes

1 Throughout this paper we suggest the INDEPTH 2013 standard reflects conditions in LMICs, since seven of the 15 countries where HDSSs are located are considered either lower middle income (India, Vietnam, Ghana, Indonesia, Côte d'Ivoire, and Papua New Guinea) or higher middle income (South Africa) according to the 2012 World Bank Classification (http://data.worldbank.org/about/country-classifications).

2 Consistent with existing standards and for comparability, we present a single standard age proportion for the under-five age group, but interested readers can find a break down into ages 0 and 1–4 in the gray shaded boxes in Tables 2 and 4.

3 For HDSSs with fewer than 5 years of data, all available years were used to calculate the numbers in Table 3.
